# Field Surgery in South Africa

**Published:** 1899-09-30

**Authors:** 


### The Hospital

A Journal of

The Medical Sciences and Hospital Administration.

Vol. XXVI.—No. 679.] Edited by Sir Henry Burdett, K.C.B., and Solomon C. Smith, M.D., M.R.C.P. [September 30, 1899.

### FIELD SURGERY IN SOUTH AFRICA.

In the only too probable event of the occurrence of hostilities in South Africa, the medical and nursing departments of the army will be expected by the public to justify the increased care which has lately been bestowed upon their organisation and equipment, and upon the conditions under which they will be required to act. If it should be found necessary, in addition to the forces now at the Cape or on their way thither, to despatch also a complete army corps of approximately fifty thousand officers and men, the principal medical officer of such a corps would be in practical command of at least twelve hundred persons, including the subordinate members of the medical staff, the nurses, the non-commissioned officers and men of the Army Service Corps, the hospital orderlies, the teamsters, and the various followers of a large hospital equipment. It would be his duty, guided by such information as commanding officers were able to impart, to keep his whole force so in hand that sufficient detachments of it might be readily available at points likely to be the scenes of armed encounters, and in such relations with the fighting line as to afford prompt assistance not only to the sick and wounded but to any men who fell out or were temporarily disabled through fatigue, sore feet, or any similar cause of incapacity. The working of the medical department is certain to be watchfully regarded at home, and to be closely, even if not severely, criticised by that section of military officers who for so many years have hindered the grant to army doctors of the distinctly military status which they have consistently asserted to be essential to the right discharge of their professional duties and obligations. On paper, at all events, and in appearance, the doctors have now received the positions and the authority which they have so long sought to obtain; and it will be incumbent upon them to give abundant proof of their fitness to occupy the positions and to exercise the authority. It will be incumbent upon them, if their actual dispositions become the subjects of hostile criticism, and if they and the men under their control are not found where they would be able quickly to disencumber the fighting line of wounded, to show that such a result was not traceable to any default of theirs, but to the nature of the information by which they were guided. The country will expect our next campaign to show that army doctors are capable of acting promptly and wisely upon their own initiative, and that the direction and control of their department may safely be committed to them in the future.

It would appear, indeed, if medical requirements are not compelled to give way before the actual exigencies of the strife, that an invasion of the Transvaal, or of the Orange Free State, might be so conducted as to exclude the greater part of the ordinary causes of camp sickness, and to give the doctors little more work than that of attention to the wounded. The invaders would be able to select their own time for advancing, and might in this way avoid many of the disease-producing hardships incidental to rain or other inclemency of weather; while the open veldt and the rocky ranges which they would have to traverse would be salubrious in the beginning, and with proper care might be preserved in this condition. The whole country is abundantly watered by numerous rivers, and, except towards the north of the Transvaal, even the valleys of these rivers are not unhealthy; while the towns, as well as the chief strategic points, are all at such elevations as to insure the absence of any dangerous amount of subsoil water. Pietermaritzburg, the lowest of these points, is more than two thousand feet, and Johannesburg, the highest of them, is nearly six thousand, above the sea. The invading force would be in no hurry, inasmuch as time would fight on their side and against their adversaries; and would be able to secure sufficient lines of communication, to arrange for the easy transport of supplies and of reinforcements, and to organise their primary and secondary bases of operations with reference to the directions in which they might be called upon to act. With these advantages, except for the bullets of the enemy, the campaign ought to be scarcely more dangerous or more wasteful of life than a period of training on Salisbury Plain; and our military authorities, whether combatant or medical, may be reasonably expected to unite their best efforts for the prevention of the sickness which has hitherto been so terrible a scourge in war. Much has already been done in this direction, but even the best records of the past should be far excelled in the future. In the Crimea, independently of men killed in action, we lost 1,761 from wounds and 16,297 from disease; 10 per cent. of the whole invading force having died from disease in the one month of January, 1855. In Egypt, in 1882, more than half the force was admitted into hospital, although from wounds and disease together there were only 79 deaths among over 13,000 men. In 1885, among 10,000 men sent to Suakin, there were only 20 per cent of admissions to hospital, and 17 deaths all told. The statistics of the expedition which was concluded by the battle of Omdurman were probably much influenced by the presence of typhoid, and we have not the figures at hand for comparison. We have a right to expect that typhoid shall not be suffered to occur, or not to attain any frequency of occurrence, at the Cape; and that a campaign there, if unhappily it should become necessary, will, we hope, show what medical skill and knowledge can accomplish in the way of preserving the health of large bodies of men engaged in actual war.

